# Fatal Crashes Among Drivers Age 60+ in Connecticut, Massachusetts, New Hampshire, and Rhode Island

**DOI:** 10.1093/geroni/igaa057.372

**Published:** 2020-12-16

**Authors:** Chae Man Lee, Beth Dugan

**Affiliations:** 1 University of Massachusetts Boston, Boston, United States; 2 University of Massachusetts Boston, Boston, Massachusetts, United States

## Abstract

Fatal crashes are related with the spatial components of physical environments (e.g. roadways, land use, and political boundaries). This study compares rates of fatal crashes among drivers age 60+ by counties in 4 states of CT, MA, NH, and RI. The GIS application is used to visualize the location of fatal crashes and to identify whether it is clustered as hotspots. This study pooled data related to fatal crashes in CT, MA, NH, and RI from the Fatal Accident Recording System (2008-2018). Sample (n=2,373) inclusion criteria were subjects (driver age 60+) had to have complete data on variables of interest and be involved in a crash with at least one fatality. More than half (n=1,387, 58.5%) of drivers had a fatal injury. Results showed that the county with the highest incidents of fatal crashes was New Haven, CT (n=183 involved, 53% fatality), Worcester, MA (n=179, 61.5% respectively), Hillsborough, NH (n=75, 65.3% respectively), and Providence, RI (n=94, 59.6% respectively). The GIS spatial analysis showed that crashes were clustered along roads with the highest speed limits (interstate highways or multilane state routes) and found that the hotspots of clustered fatal crashes were located in counties with big cities with high population densities (New Haven CT, Hartford CT, Springfield MA, Worcester MA, Boston MA, Concord NH, and Providence RI). Identification of these crash hotspots will be beneficial for drivers and policy makers. The findings may alert drivers to high risk areas and policy makers can implement countermeasures.

